# Small-molecule antagonist of VLA-4 (GW559090) attenuated neuro-inflammation by targeting Th17 cell trafficking across the blood-retinal barrier in experimental autoimmune uveitis

**DOI:** 10.1186/s12974-021-02080-8

**Published:** 2021-02-18

**Authors:** Yi Hsing Chen, Malihe Eskandarpour, Xiaozhe Zhang, Grazyna Galatowicz, John Greenwood, Sue Lightman, Virginia Calder

**Affiliations:** 1 UCL Institute of Ophthalmology, University College London, 11-43 Bath Street, London, EC1V 9EL UK; 2 Department of Ophthalmology, Chang Gung Memorial Hospital, Taoyuan, Taiwan; 3 College of Medicine, Chang Gung University, Taoyuan, Taiwan; 4 Moorfields Eye Hospital and UCL Biomedical Research Centre, London, UK; 5 Moorfields Eye Hospital, London, UK

**Keywords:** Integrin (α4β1, VLA-4), Uveitis, Experimental autoimmune uveitis, Leukocyte migration, Th17 cells, Inflammatory monocytes/macrophages

## Abstract

**Background:**

The integrin VLA-4 (α4β1) plays an important role in leukocyte trafficking. This study investigated the efficacy of a novel topical α4β1 integrin inhibitor (GW559090, GW) in a mouse model for non-infectious posterior uveitis (experimental autoimmune uveitis; EAU) and its effect on intraocular leukocyte subsets.

**Methods:**

Mice (female; B10.RIII or C57Bl/6; aged 6–8 weeks) were immunized with specific interphotoreceptor retinoid-binding protein (IRBP) peptides to induce EAU. Topically administered GW (3, 10, and 30 mg/ml) were given twice daily either therapeutically once disease was evident, or prophylactically, and compared with vehicle-treated (Veh) and 0.1% dexamethasone-treated (Dex) controls. Mice were sacrificed at peak disease. The retinal T cell subsets were investigated by immunohistochemistry and immunofluorescence staining. The immune cells within the retina, blood, and draining lymph nodes (dLNs) were phenotyped by flow cytometry. The effect of GW559090 on non-adherent, adherent, and migrated CD4^+^ T cell subsets across a central nervous system (CNS) endothelium was further assayed in vitro and quantitated by flow cytometry.

**Results:**

There was a significant reduction in clinical and histological scores in GW_10_- and Dex-treated groups as compared to controls either administered therapeutically or prophylactically. There were fewer CD45^+^ leukocytes infiltrating the retinae and vitreous fluids in the treated GW_10_ group (*P* < 0.05). Immunofluorescence staining and flow cytometry data identified decreased levels of retinal Th17 cells (*P* ≤ 0.001) in the GW_10_-treated eyes, leaving systemic T cell subsets unaffected. In addition, fewer Ly6C^+^ inflammatory monocyte/macrophages (*P* = 0.002) and dendritic cells (*P* = 0.017) crossed the BRB following GW_10_ treatment. In vitro migration assays confirmed that Th17 cells were selectively suppressed by GW559090 in adhering to endothelial monolayers.

**Conclusions:**

This α4β1 integrin inhibitor may exert a modulatory effect in EAU progression by selectively blocking Th17 cell migration across the blood-retinal barrier without affecting systemic CD4^+^ T cell subsets. Local α4β1 integrin-directed inhibition could be clinically relevant in treating a Th17-dominant form of uveitis.

**Supplementary Information:**

The online version contains supplementary material available at 10.1186/s12974-021-02080-8.

## Background

Non-infectious posterior uveitis is an inflammatory disease targeting the uveal tract in the eye  mediated by leukocytes that infiltrate the retinal tissues. It has the potential to threaten vision and accounts for 10–15% of patients who are blind, especially affecting people of working age [1, 2]. It is considered to be an immune-mediated disorder based on clinical treatment responses to corticosteroids and immunosuppressants, the ability of adoptively transferred retinal antigen-specific CD4^+^ T cells to induce an experimental model of autoimmune uveitis (EAU), and histology obtained from postmortem uveitis eyes [3, 4]. The process is accompanied by evidence of blood-retinal barrier (BRB) breakdown [5]; leukocytes infiltrating the anterior and/or posterior chamber and vitreous; immigration of retinal CD4^+^ T cells, mainly Th1 (secreting IFNγ and expressing Tbet) and Th17 (secreting IL-17A and expressing RORγt) [6]; and structural damage mediated via cytokines released by infiltrating leukocytes, including activated macrophages and neutrophils [7].

Recruitment of effector CD4^+^ T cells to an immune privileged site involves co-ordination of adhesion molecules expressed by circulating leukocytes and by endothelial cells within the blood vessels [8, 9]. The specific cell migration process is largely mediated by leukocyte functional antigen-l (LFA-1)/intercellular adhesion molecule-1 (ICAM-l) and very late activation antigen-4 (VLA-4)/vascular cell adhesion molecule-1 (VCAM-1) interactions [10, 11]. There is evidence of increasing expression of ICAM-1 and VCAM-1 in studies of human uveitis retinal specimens and from EAU retinal tissues [5, 9] and enhanced VCAM-1 expression specifically in focal areas of the retinal post-capillary venules when inflammation is initiated and when EAU is evident [5, 12]. However, in vitro studies suggested that lymphocyte migration is more dependent on LFA-1/ICAM-1 than VLA-4/VCAM-1 interactions [13, 14]. The discrepancy may be due to the fact that the VLA-4/VCAM-1 axis participated in migration only under inflamed conditions [13]. Indeed, targeting VLA-4 or LFA-1 molecules systemically has previously been shown to barricade lymphocytes from crossing the BRB and ameliorate the clinical signs of EAU, indicating the importance of the VLA-4/VCAM-1 pathway in inflamed retinae [15–20]. Whether there is a preferential use of specific integrins by Th1 cells versus Th17 cells has not been addressed within the uveitogenic CD4^+^ T cell subsets.

The side effects of systemically administered integrin inhibitors have been reported with long-term use, for example, infection and progressive multifocal leukoencephalopathy (PML) in Natalizumab (humanized α4 antibody) treatment in multiple sclerosis patients [21]. Recently, a topical α4β1 (VLA-4, CD49d/CD29) integrin inhibitor (GW559090) was shown to be clinically efficacious in a mouse model of dry eye disease [22–24]. It has been reported to exert its clinical effect through its high affinity binding to the α4β1/VLA-4 integrin and specific blockade of cell interaction to VCAM-1 [22]. In this study, we investigated the effect of topically applied GW559090 in EAU, and the potential cellular mechanisms involved.

## Material and methods

### EAU induction and treatment

Female B10.RIII mice, aged 6 to 8 weeks, were obtained from an in-house colony and female C57Bl/6 mice were purchased from Charles River Laboratories (*n* = 5–8 per experimental group). All studies were conducted according to the UK Home Office Regulations on the Care, Welfare and Treatment of Laboratory Animals and in compliance with the Association for Research in Vision and Ophthalmology (ARVO) Statement on the Use of Animals in Ophthalmic and Visual Research.

EAU was induced by immunization of naïve mice with subcutaneous 400 μg human IRBP peptides (IRBP_161–180_ for B10.RIII mice; IRBP_1–20_ for C57BL/6 mice) in complete Freund’s adjuvant (CFA; Sigma, Gillingham, UK; 1:1 vol/vol) supplemented with 1.5 mg/ml *Mycobacterium tuberculosis* H37 Ra (Difco Microbiology, Voigt Global Distribution, Lawrence, KS, USA). All mice simultaneously received 0.4 μg *Bordetella pertussis* toxin (Sigma-Aldrich, UK) intraperitoneally. Mice receiving CFA in phosphate-buffered saline (PBS) alone were used as controls.

Eyedrops (5 μL) containing α4β1 integrin antagonist GW559090 ((S)-3-(4-((4-carbamoylpiperidine-1-carbonyl)oxy)phenyl)-2-((S)-4-methyl-2-(2-(otolyloxy)acetamido)pentanamido) propanoic acid, kindly provided by GSK) solubilized in PBS (pH = 7) were given twice daily in both eyes either prophylactically (from days 0 to 14 post EAU induction) or therapeutically (from days 10 to 18 post EAU induction). The concentrations of GW559090 were selected based on previously published data [22, 23]. GW559090 was solubilized in PBS and used at a final concentration of 3 mg/ml (GW_3_), 10 mg/ml (GW_10_), or 30 mg/ml (GW_30_) and compared to vehicle-treated controls (Veh) or 0.1% dexamethasone eye drops (Maxidex®, Alcon, UK; Dex) as treatment controls. In some experiments, 10 mg/ml GW559090 was applied to the right eye only (GW_10R_) to evaluate whether it had a sympathetic effect on the untreated left eye (GW_10L_). Mice were sacrificed for further investigation when those in Veh group reached peak disease (day 14 in B10.RIII mice, day 18–21 for C57BL/6 mice).

### Disease grading

Dilated ocular examination was performed using retinal fundoscopy (Micron III; Phoenix Research Laboratories, Pleasanton, CA, USA) on days 10, 14, and/or day 18–21 after disease induction. Pupils were dilated with topical 2.5% phenylephrine and 1% tropicamide, and the corneas protected with 0.2% carbomer eye gel (Bausch & Lomb Ltd, UK). The clinical grading was performed by two independent experts [25]. In summary, optic disc neuropathy, vasculitis, retinitis, and structural damage were scored separately in each eye, from 0 (no disease) to 5 (severe inflammation) with half-point increments and then scores were summed to generate the clinical score of each eye on a scale from 0 to 20. The clinical score attributed to each mouse corresponds to the mean of the scores from both eyes.

### Immunohistochemistry staining for CD45^+^ cells

Eyes were collected and fixed in situ with 4% glutaraldehyde for 1 h, followed by overnight fixation in 10% formalin, and embedded in paraffin (Sigma-Aldrich, UK). A series of four anterior-posterior sections passing through the optic nerve with 4-μm-thick sections were prepared and stained with hematoxylin and eosin (H&E). The results were graded by two independent, masked observers [26].

For immunohistochemistry staining, the sections were blocked with 5% goat serum in PBS for 1 h at room temperature and then incubated with primary rabbit polyclonal antibody to CD45 (1:200; ab10558; Abcam, Oxford, UK) at 4 °C overnight. After washing, the sections were incubated with biotinylated anti-rabbit IgG Ab (Vectastain Elite ABC HRP Kit, Abcam, UK) for 1 h followed by visualization with a brick-red indicator product (NovaRED™, Vector Laboratories, UK). Stained sections were scored [27, 28]. In brief, cellular infiltrates (0–30) were scored within the ciliary body, vitreous, vessels, rod outer segments, and choroid, whereas the structural score (0–12) was based on the rod outer segments, neuronal layers, and retinal morphology. Both scores were added for a final score for each eye, and mean scores from both eyes calculated for each mouse (0–42).

### Immunofluorescence staining locating immune cells within the eye

Ocular anterior-posterior sections were blocked with 5% bovine serum albumin (BSA) in TBS for 2.5 h, then incubated overnight at 4 °C with the following primary antibodies in different combinations: CD4 (rat; 1:100; Santa Cruz Biotechnology, USA), Tbet (rabbit; 1:100; Santa Cruz Biotechnology, USA), RORγt (goat; 1:100; Santa Cruz Biotechnology, USA), FoxP3 (rabbit; 1:100; Abcam, USA), and Iba-1 (myeloid cell marker; mouse; 1:100; Santa Cruz Biotechnology), diluted in TBS supplemented with 1% BSA. After three washes in TBS, sections were incubated in the dark for 2 h with species-specific secondary antibodies coupled to different fluorochromes as indicated in the data: Alexa Fluor 488, 555, and 633-conjugated secondary antibody (1:200; Molecular Probes, Eugene, OR, USA). After three final washings in PBS (20 min each), sections were mounted with Vectashield antifade mounting medium containing DAPI (Thermo Fisher Scientific, UK).

Images from at least three separate fields per retinal section (× 400) were captured using a Zeiss LSM 700 (or 710) confocal microscope. Staining patterns were observed to be consistent between consecutive single-, double-, or triple-stained sections using ZEN 2.1 software (Carl Zeiss, Oberkochen, Germany). ImageJ software (http://imagej.nih.gov/ij/) was used for counting cells in the images. These images were used for counting populations of CD4^+^Tbet^+^, CD4^+^RORγt^+^, CD4^+^Tbet^+^RORγt^+^, CD4^+^Foxp3^+^, and Iba-1^+^ cells in eye tissue. For cell scoring within the anterior chamber, total cells from three non-overlapping images were counted; for scoring cells in inner retina and vitreous, cell counts were from 5 images to cover the whole eye. Cells were verified with DAPI and counted within the anterior chamber, ciliary body, vitreous, vessels, retinal layers and choroid, and were summed up for a final score. The mean percentages of CD4^+^ T cell subsets in different experimental groups were calculated.

### Identification of T cell subsets and myeloid populations by flow cytometry

Single-cell suspensions were prepared from retinal tissues, draining lymph nodes (dLNs) and blood and investigated by flow cytometric staining for their expression of CD4-FITC (RM4-5) and CD11b-PE (M1/70) (myeloid cell marker; eBioscience); CD11c-BV786 (HL3), Ly6G-PE-CF594 (IA8), CD45-BV605 (30-F11), FoxP3-PE/Cy5 (FJK-16 s), Ly6C-PerCP/Cy5.5 (HK 1.4), IFNγ-PE-Cy7 (XMG1.2), IL-17A-APC (TC11-18H10.1), and CD64-BV421 (X54-5/7.1; all BD Biolegend). In experiments investigating the myeloid population, data were obtained from 2 to 3 eyes pooled from the same treatment group and at least 10,000 events were acquired in order to have sufficient cell numbers for analysis. For intracellular cytokine staining, cultures were treated for 4 h with PMA (50 ng/mL) and ionomycin (1 μg/mL), adding brefeldin A (1 μg/mL; all Sigma-Aldrich) for the final 1 h. Cells were incubated with relevant surface antibodies and a Live/Dead fixable dye (Molecular Probes; Life Technology, Paisley, UK) for 30 min at 4 °C, before resuspending in FACS buffer and fixed. Cells were then permeabilized using fixation/perm buffer (Fix and Perm cell permeabilization kit; eBioscience, La Jolla, CA, USA) for 10 min. Transcription factor expression was determined using the Foxp3 cell Staining Kit (BD Biosciences), according to the manufacturer’s instructions. Relevant isotype control mAbs (BD Biosciences and eBioscience) were combined as fluorescence minus one (FMO) controls. Up to one million live cells per sample were acquired on a BD FACSCalibur using CellQuest (BD Cytometry Systems, Oxford, UK) or a BD LSR Fortessa-x20 using FACSDiva (BD Cytometry Systems). Isotypes and FMO controls were used for accurate gating. Compensation matrices were performed using OneComp Beads (eBioscience, UK). Flow cytometric data were analyzed using FlowJo software (Version 9.6.4; Tree Star). Debris and doublets were excluded, and live cells were gated before further analysis.

### In vitro leukocyte migration assays

Human microvascular endothelial cells (HMEC; a gift from Professor P. Turowski UCL Institute of Ophthalmology, London, UK) were cultured with EGM2-MV (Endothelial cell growth medium-2 bullet kit, Lonza, USA) with supplements and the culture medium replaced weekly. Transmigration of lymphocytes was assayed using 6.5-mm Transwell inserts with a 3-μm pore size in a 24-well plate (Corning, Sigma-Aldrich, UK). HMEC cells were seeded on fibronectin-coated filters at a density of 3 × 10^5^ cells and stimulated with human IFNγ (200 IU/ml, PeproTech, UK) 3 days before the assay [29].

Venous blood was obtained from healthy volunteers and collected in EDTA (3 mmol/L). Human buffy coat leukocytes were isolated by density gradient centrifugation by layering over Histopaque (Sigma-Aldrich, UK) and centrifugation at 300×*g* to obtain mononuclear leukocytes. Mononuclear lymphocytes were cultured at 1 × 10^6^/ml in T cell medium (RPMI 1640 supplemented with 10% FCS, 100 U/ml penicillin, 100 mg/ml streptomycin, 1 mM sodium pyruvate, 1 mM nonessential amino acids, 2 mM L-glutamine, and 50 μM β-mercaptoethanol (all Sigma-Aldrich)). Lymphocytes were stimulated weekly with phytohemagglutinin (PHA; 3 μg/ml) and human rIL-2 (50 IU/ml; PeproTech, UK) added every 3 days for a week before the assay [30], and lymphocytes were re-stimulated with PHA 24 h before the assay. This methodology supports the maintenance of  the effector T cells in culture for a maximum of 4 weeks, after which their viability is reduced [31, 32]. Cells were pre-treated with 10, 100, or 1000 μg/ml GW559090 or saline (Sal) as controls for 2 h prior to assay.

To assess migration, 2 × 10^6^ lymphocytes were washed by centrifugation, re-suspended in 100-μl fresh T cell medium and placed into each Transwell insert. The lower wells were filled with 500 μl of T cell medium. Following incubation for 18 h at 37 °C, not adherent, adherent and migrated lymphocytes were carefully harvested from each compartment and stained for CD4-FITC (OKT4), RORγt-APC (AFKJS-9), Tbet-PE (eBio4B10) (all from eBioscience), and FoxP3-BV711 (236AIE7; BD Biosciences) and acquired for flow cytometry, as described above. In some experiments, additional markers for CXCR3-BUV395 (1C6/CXCR3), CCR6-BV421 (G034E3), IFNγ-BV605 (4S.B3; BD Biosciences), and IL-17A-PE (eBio64DEC17; eBioscience) were used. BD Liquid Counting Beads (BD Biosciences) was used to evaluate the cell numbers within each chamber, according to the manufacturer’s protocol.

### Statistics

Results are presented as the mean ± SEM for at least 5 data points and mean ± SD for 3 data points. For comparing means from two groups, *P* values were calculated by the unpaired two-tailed Student *t* test. For analysis of the low frequency myeloid data, Bonferroni-multiple *t* tests were used. For comparison of means from three or more groups for two factors, two-way ANOVA was used to reveal differences in the data sets, followed by Dunn’s or Tukey’s multiple comparison post hoc test. Statistical analyses were performed using GraphPad Prism 6 software. *P* values < 0.05 were considered statistically significant.

## Results

### Topical VLA-4 inhibitor prevented retinal inflammation

**Fig. 1:**
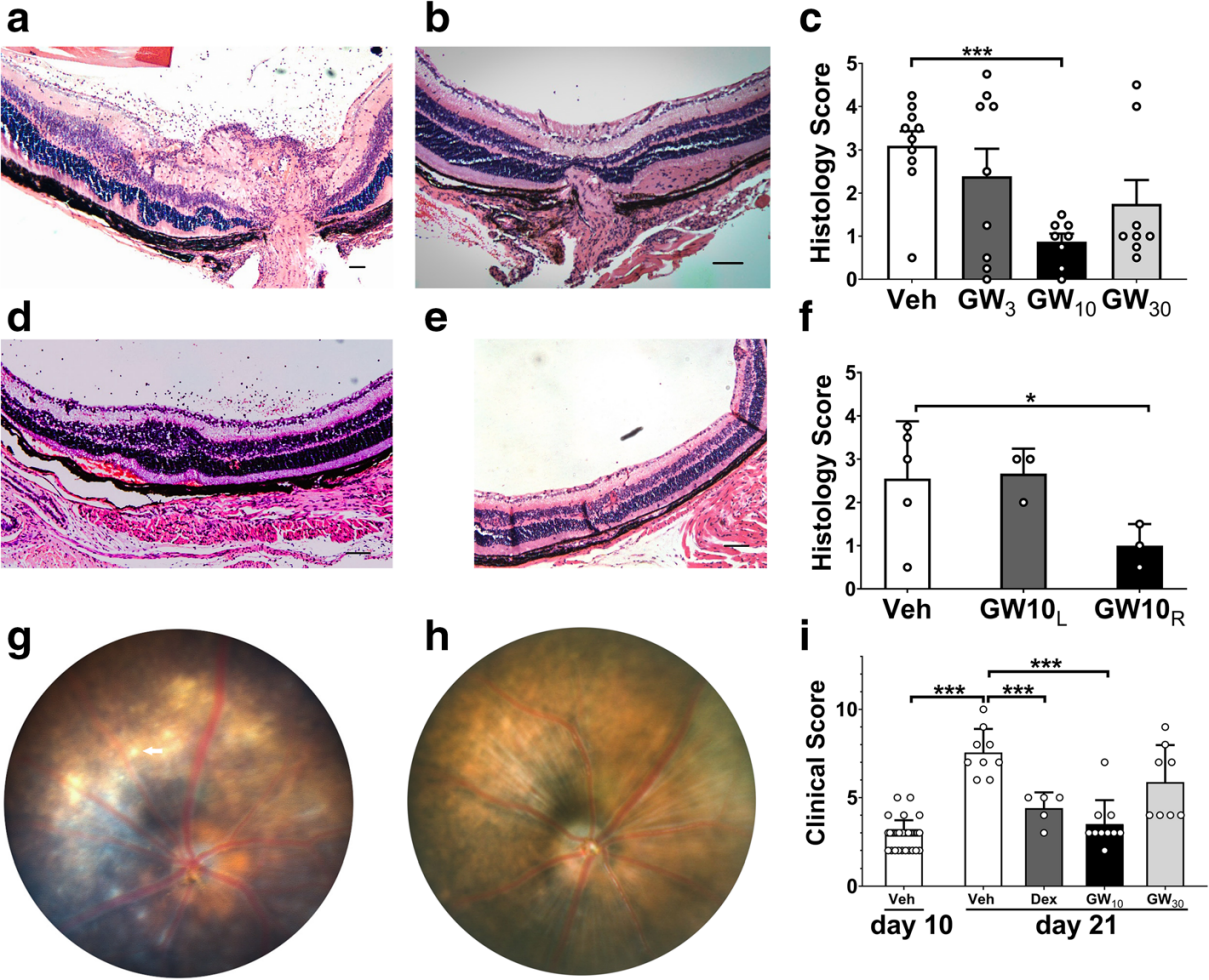
Topical administration of α4β1 VLA-4 inhibitor either at the time of EAU induction or when disease was evident (10 days post EAU induction) demonstrated a reduction of cellular inflammation in the EAU eyes. **a**, **b** H&E staining of eye sections including optic nerve illustrating increased numbers of cells within the vitreous, retinal folding, and disruption of the retinal layers in vehicle-treated (Veh) mice (**a**) than the 10 mg/ml GW559090 (GW_10_)-treated eye (**b**). **c** Gradings of histological scores for EAU eyes treated with GW_3_, GW_10_, and GW_30_ and vehicle controls. **d**, **e** H&E staining of retinal sections of GW_10_ untreated left eye (**d**, GW_10L_) and treated right eyes (**e**, GW_10R_) demonstrating decreased retinal folding and fewer infiltrating cells in the treated eye. **f** Comparing the histological scores for EAU mice treated preventively with 10 mg/ml of GW559090 in right eye only and the untreated left eye. **g** Retinal fundus imaging of EAU eyes at peak stage of disease. **h** There were decreased peri-vascular infiltrates, disc infiltration, subretinal infiltrations, and vasculitis in GW_10_ therapeutic treatment in EAU. **i** Clinical scores after EAU signs were evident by day 10 post immunization (d10pi) and after a further 7 days’ topical treatment with GW559090. The mice developed clinical extensive disease in Veh group, while in GW_10_ and Dex treatment group, a significant reduction of clinical severity was observed. Values were mean ± SEM for 5 or more data points (**c**, **i**) and mean ± SD for 3 data points in **f**. *n* = 8 in preventive treatment experiment (**a**–**e**), *n* = 3 in unilateral eye treated group (**f**), and *n* = 5 in therapeutic treatment experiment (**g**–**i**). Scale bar = 100 μm. *P < 0.05, ***P < 0.001 using a two-tailed, unpaired Student’s *t* test

The GW559090-treated B10.RIII EAU mice demonstrated a reduction of retinal inflammatory infiltrates as compared to vehicle-treated (Veh) controls at peak disease. Retinae from Veh group became clinically inflamed and showed moderate to severe signs of EAU in histopathologic examination by H&E staining on day 14. For example, retinal structural damage, retinal vasculitis, optic neuropathy, and diffuse infiltration of cells into the vitreous, uvea, and retina (Fig. 1a, b). In contrast, mice treated with GW559090 topically from day 0 to 14, showed minimal disease development in the GW_10_ group (*P* = 0.005; Fig. 1d, e). The reduction of EAU severity was significant in the GW_10_ group compared to Veh group, but the effect was not seen in GW_3_ and GW_30_ groups. In addition, there was a significant decrease in disease incidence in GW_10_ group (*P <* 0.05; Fig. 1c). In mice treated in just the right eye, disease was suppressed in the treated eye (GW_10R_), but EAU continued to develop in the contralateral eye (GW_10L_; Fig. 1f).

### Topical VLA-4 inhibitor given therapeutically arrested disease progression

EAU in C57BL/6 mice was induced as described above. All mice were assessed for their development of EAU at day 10 following disease induction and were confirmed to have a similar disease severity (2.9 ± 0.1). After treating the mice for 7 days, Veh groups were compared with GW_10_, GW_30_, and Dex treatment. There was a decrease in disease progression in the GW_10_ group (3.5 ± 0.5, *P* < 0.0001) and the Dex group (4.4 ± 0.4, *P* < 0.0001) as compared to Veh group (7.6 ± 0.4; Fig. 1g, h). A decrease in disease scores involving optic disc infiltrates, subretinal infiltrates, and peri-vascular infiltrates was observed in GW_10_ group, and there was a reduced clinical severity comparable to the Dex-treated group (Fig. 1h). The results were further confirmed by H&E staining (data not shown).

### Cellular infiltration into the retina was preferentially blocked by GW559090

**Fig. 2:**
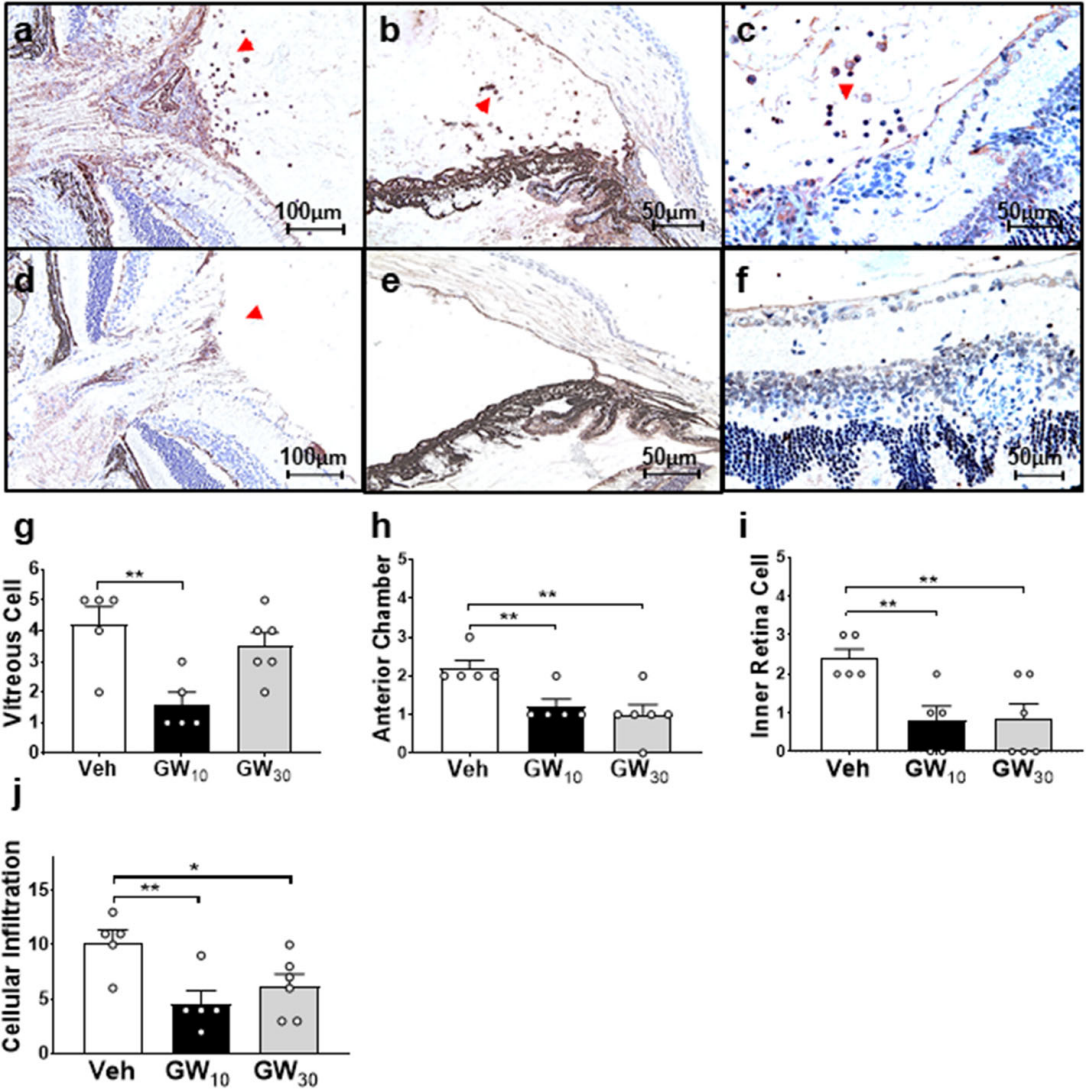
Localization of leukocytes within the eyes after treating with GW559090. There was an increase in leukocytes around the optic nerve (× 200; **a**), within the anterior chamber (× 200; **b**), retinal layers, and within the vitreous cavity (× 400; **c**) in vehicle-treated control (Veh). In GW_10_-treated eyes, decreased levels of cells were detected around the optic nerve (× 200; **d**), in the anterior chamber (× 200; **e**), and within the vitreous (× 400; **f**). Mean ± SEM scores were calculated for cell numbers infiltrating the vitreous (**g**), anterior chamber (**h**), and inner retina (**i**) in all groups. **j** Cellular infiltration score. *n* = 5 in each treatment group. *P < 0.05; **P < 0.01, using a two-tailed, unpaired Student’s *t* test

By staining retinal sections with CD45, the infiltrating leukocytes were able to be analyzed quantitatively by their location. In EAU controls, leukocytes were localized in the anterior chamber, optic nerve, vitreous cavity, and retinal layers (Fig. 2a–c). There were fewer leukocytes around the optic nerve head, in the vitreous, anterior chamber, and inner retina following GW_10_ treatment (Fig. 2d–f) and levels were significantly decreased relative to Veh controls (Fig. 2g–i). There was an overall decrease in cell numbers within the anterior chamber and inner retinal layer in all treated groups (Fig. 2h, i). In summary, a significantly reduced cellular infiltration score was observed in both GW_10_ and GW_30_ groups (Fig. 2j), although some retinal folding remained (Fig. 2f), and the structural damage scores in all groups were relatively low and did not differ (data not shown). Despite cellular infiltration scores being reduced in the GW_30_ group (Fig. 2j), the cumulative disease scores revealed a significant disease reduction only in the GW_10_ group (data not shown).

### Topical VLA-4 inhibitor targeted retinal Th17 cell infiltration in vivo

**Fig. 3:**
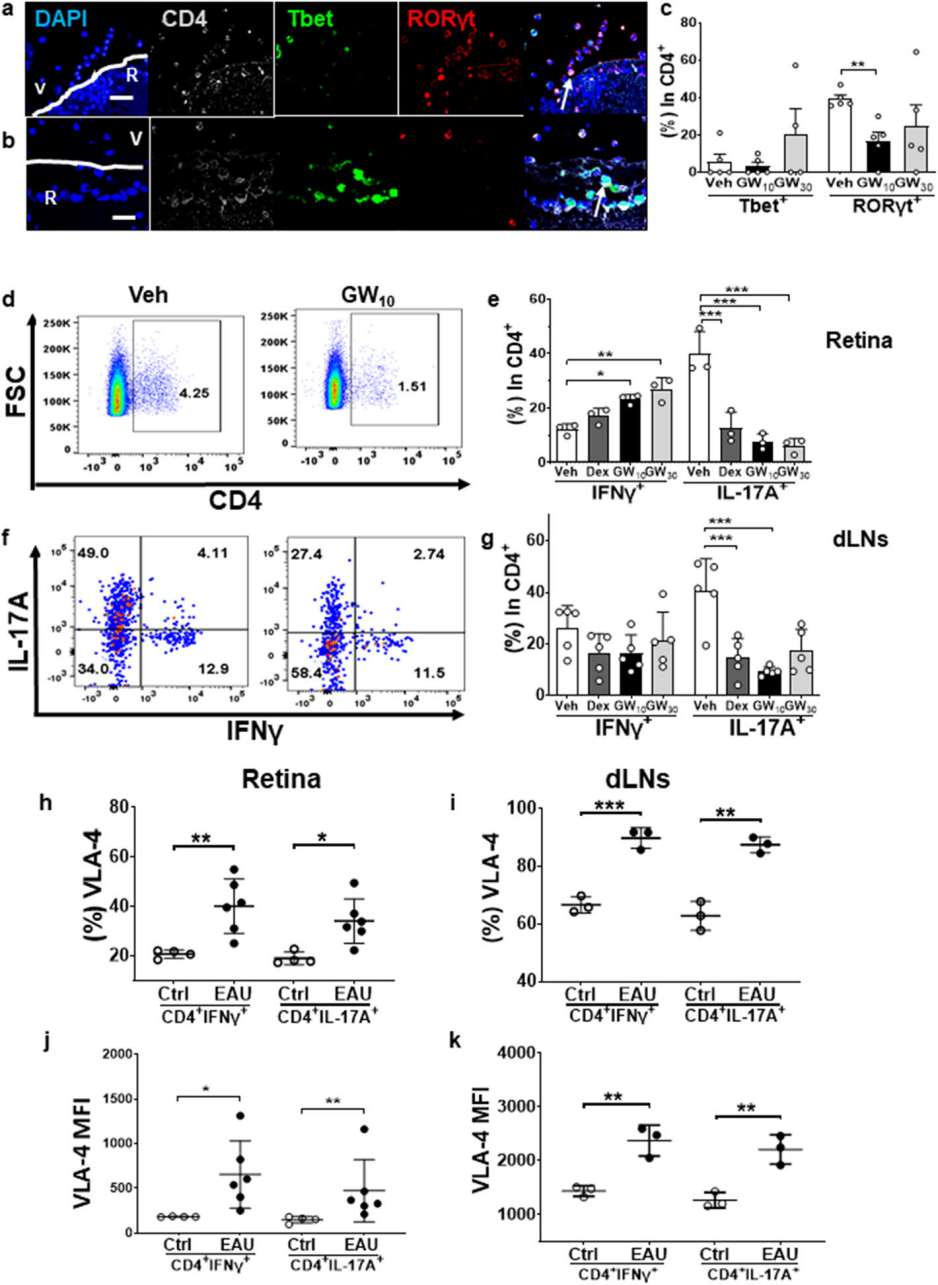
Immunofluorescence staining and flow cytometric analysis of CD4^+^ T cell subsets within the retina after GW559090 treatment. **a** There were both Th1 (CD4^+^Tbet^+^) and Th17 (CD4^+^RORγt^+^) cells in the vitreous and inner retinal layers in Veh group. **b** In GW_10_-treated group, there were decreased numbers of CD4^+^ T cells infiltrating the retina which were mainly Th1 cells. **c** Counting the CD4^+^ T cell subsets within the vitreous revealed that Th17 cells were mostly suppressed by 10 mg/ml GW559090 treatment. **d** Representative retinal flow cytometric figures for CD4^+^ T cells. **e** Identification of CD4^+^ T cell subsets in the retina by expression of IFNγ or IL-17A. **f** Representative retinal flow cytometric figures for the distribution of IFNγ or IL-17A within CD4^+^FoxP3^-^ live cells. **g** Figures represent the results of flow cytometric analysis for CD4^+^ T cell subsets within the dLNs. **h**, **i** VLA-4 expression in CD4^+^ T cell subsets within the retina (**h**) and dLNs (**i**). **j**, **k** Mean fluorescence intensity (MFI) of VLA-4 expression in Th1 and Th17 cells in eye (**j**) and dLNs (**k**). Means ± SD. *n* = 5 in **c** and *n* = 3 in **e** and **g**–**k** in each treatment group. Eye data were from single eyes in individual mice. Data were from one experiment which is representative of three separate experiments that were performed. *P < 0.05; **P < 0.01; ***P < 0.001 compared to Veh group, using two-tailed, unpaired Student’s *t* test

There was a mixture of Th1 (CD4^+^Tbet^+^) and Th17 (CD4^+^RORγt^+^) cells, with Th17 cells being the predominant subset in EAU retinae (Veh group, Fig. 3a). These cells were mostly localized to the inner retinal layer and vitreous (Fig. 3a). In contrast, in the GW_10_-treated group, there were fewer cells detected in the posterior chamber of the eye, and the dominant CD4^+^ T cell subset was Th1. Most cells were located within the retina and there were fewer infiltrating cells observed in the vitreous cavity (Fig. 3b). There were minimal levels of CD4^+^FoxP3^+^ regulatory T cells (Tregs) in the vitreous and posterior chamber in Veh control eyes and some Treg cells were detected in the anterior chamber, vitreous, and inner retina in the GW_10_ eyes (data not shown). By calculating all of the CD4^+^ T cell subsets within vitreous by immunofluorescence staining of sections, Th17 cells were mostly suppressed by treatment with GW_10_, while the proportion of Th1 cells was unaffected (Fig. 3c).

The effects of GW559090 treatment on different T cell subsets were further validated by single-cell analysis in the retinae, blood and dLNs by flow cytometry, investigating cytokine expression by Th1 (CD4^+^IFNγ^+^), Th17 (CD4^+^IL-17A^+^) in CD4^+^FoxP3^-^ live cell region (Fig. 3d and supplementary Fig. 1a). The results confirmed the observations from immunofluorescence staining, that Th17 cells were predominant in Veh eyes (Fig. 3e). Within the CD4^+^ T cell subsets, there was a decrease of Th17 cells in the GW_10_-treated group (7.6 ± 1.7%, *P* < 0.001) and the GW_30_-treated group (5.9 ± 1.6%, *P* < 0.001) and there were increased levels of Th1 cells in both the GW_10_-treated group (21.0 ± 4.8%, *P* = 0.018) and the GW_30_ group (22.7 ± 3.1%, *P* = 0.004) inside the eye as compared to Veh group (Fig. 3e). There were no significant changes in percentages of Treg cells following topical application of GW559090 in the eye (data not shown). In the draining LNs, a significantly reduced level of Th17 cells in GW_10_ and Dex groups compared to Veh group was identified, but not for other CD4^+^ T cell subsets (Fig. 3f–g). In addition, there was no significant change regarding Th1 and Th17 cells in the blood from mice treated topically with GW559090 (data not shown). There was an increase of Tregs in GW_10_, GW_30_, and Dex group in the blood compared to controls but this effect was not found in the retinal cells (data not shown).

### Increased VLA-4 expression on all retinal CD4^+^ T cell subsets in EAU

VLA-4 expression and the mean fluorescence intensity were increased in all CD4^+^ T cell subsets in EAU than controls in retinae and dLNs (Fig. 3h–k and supplementary Fig. 2) but not in blood (data not shown). The mean fluorescence intensity of cells expressing VLA-4 molecules on their surface did not significantly differ between Th1 and Th17 in EAU lymphocytes in both retinae and dLNs.

### Inflammatory retinal monocytes/macrophages were blocked by GW559090 in EAU

**Fig. 4:**
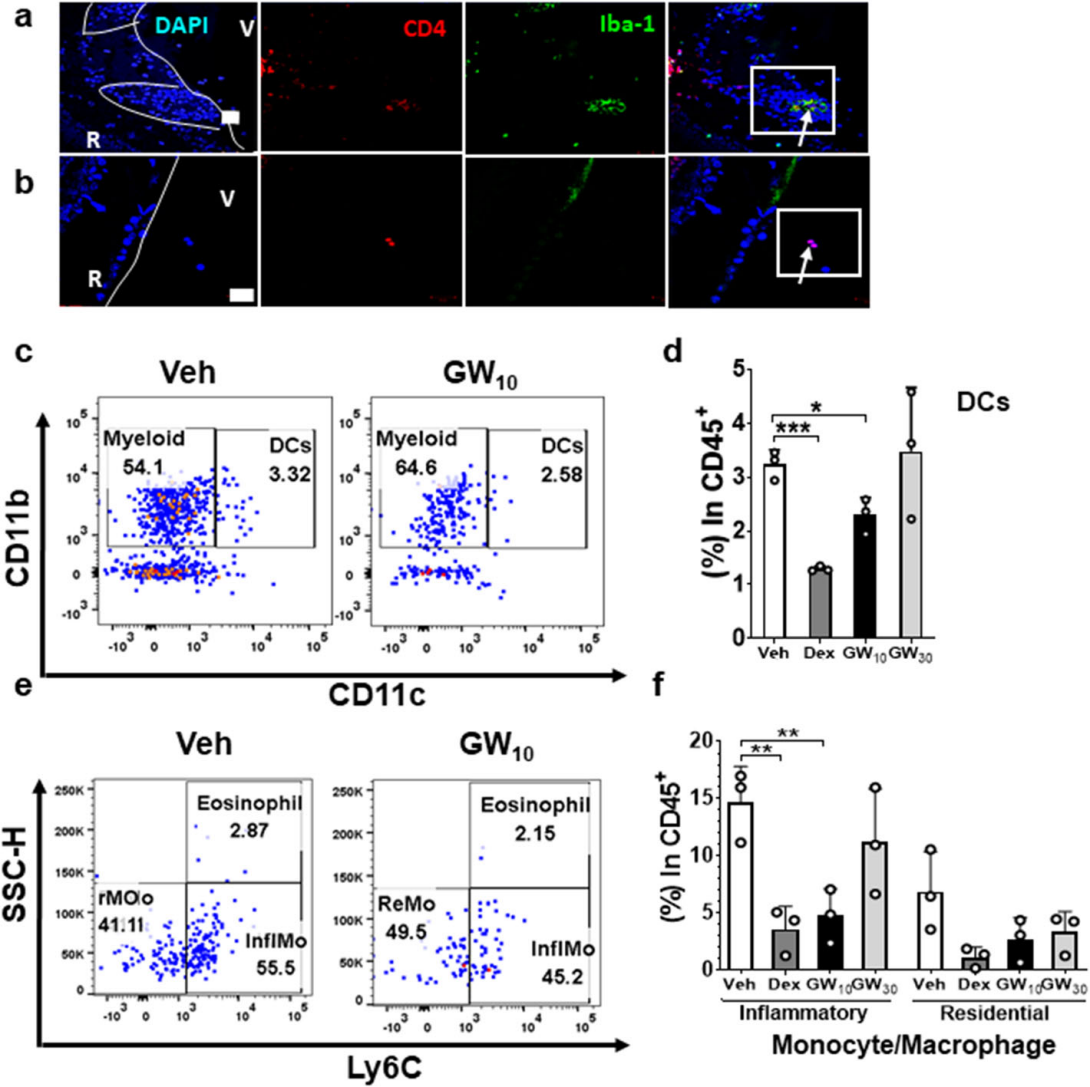
Localization and effect of GW559090 treatment on myeloid cells. **a** There were more myeloid cells (CD4^-^Iba-1^+^ cells) present within the inner retinal (R) layers in Veh group (× 400). **b** There were reduced levels of CD4^+^ T cells observed within the vitreous (V) and only very limited numbers of myeloid cells in the eyes of GW_10_ group (× 400). **c** Representative flow cytometry figure depicting CD11b^+^CD11c^-^ myeloid population and CD11b^+^CD11c^+^ dendritic cells (DC) from CD45^+^ cell gates in single live cells. **d** Level of DCs in each treatment group illustrating a decrease of DCs in the eyes treated with Dex and GW_10_ compared to the control. **e** Representative flow cytometry figures illustrating inflammatory monocytes/macrophages (Ly6C^+^) and resident monocytes/macrophages (Ly6C^-^) from CD45^+^CD11b^+^CD11C^-^CD64^+^L6G^-^ single live cell gate. **f** Comparing percentages of inflammatory and resident monocytes/macrophages between treatment groups (**d**, **f**). *n* = 3 data points per group from pooled two eyes for flow cytometric analysis. Data were from a single experiment which is representative of three experiments that were performed. Mean ± SD. *P < 0.05, **P < 0.01, ***P < 0.001 using a two-tailed unpaired Student’s *t* test

Since VLA-4 is also expressed by myeloid cells, the effects of GW559090 on myeloid cell expression levels were further analyzed by immunofluorescence staining using a panel of CD4, Iba-1, and DAPI. In the control Veh group, CD4^+^ T cells were increased within the vitreous cavity and retinal layers and Iba-1^+^ myeloid cells were mainly located in the inner retinal vascular areas (Fig. 4a). In GW_10_ group, of the few infiltrating cells, most were CD4^+^ T cells rather than myeloid cells (Fig. 4b).

Inflammatory and resident monocyte/macrophages were also investigated by flow cytometry, comparing cells isolated from retinal tissues and blood (Supplementary Fig. 1b) [33]. In the eyes, a decrease in myeloid cells (CD45^+^Ly6G^-^CD11b^+^CD11c^-^CD64^+^) was only detected in the Dex group (*P* = 0.027) but not in any of the GW559090-treated groups (data not shown). There was a decrease of CD45^+^CD11b^-^CD11c^+^ dendritic cells (DCs) in the retina in Dex and GW_10_ group (*P* = 0.0002 and 0.017, respectively, Fig. 4c, d). Within the myeloid population, however, there were decreased levels of CD45^+^Ly6G^-^CD11b^+^CD11c^-^CD64^+^Ly6C^+^ inflammatory monocytes/macrophages, while levels of Ly6C^-^ resident monocytes/macrophages within the eyes were unchanged after GW_10_ treatment (Fig. 4e, f). There were very few eosinophils in the retinae and their presence did not alter with treatment. Similarly, GW treatment did not affect levels of neutrophils or NK cells in the eye (data not shown). In the blood, topical treatments did not have any effect on levels of activated monocytes or CD45^+^CD11b^-^CD11c^+^ DCs (data not shown). Systemic neutrophils were decreased in the Dex group but were unaffected by GW559090 (data not shown).

### Adhesion of Th17 cells was decreased by GW559090 in vitro

**Fig. 5:**
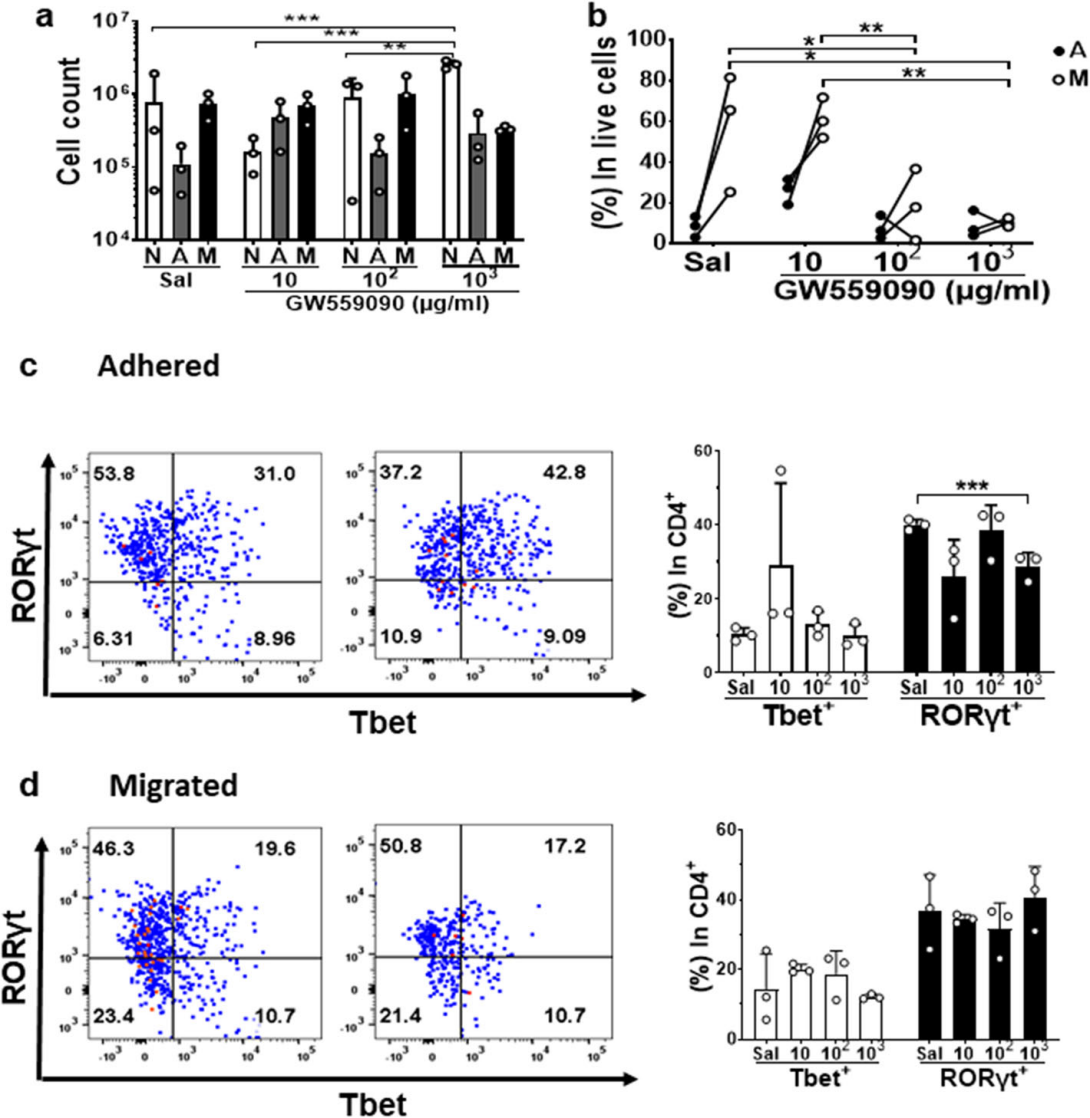
Effect of GW559090 on human PBMCs migration and CD4^+^ T cell subsets in vitro. **a** The leukocyte cell number in non-adherent (N), adhered (A), and migrated (M) populations in saline-treated controls (Sal) and 10, 10^2^, and 10^3^ μg/ml of GW559090-treated groups. **b** Percentages of live cells adhered and migrated in each group. **c**, **d** CD4^+^ T cell subsets analyzed by Tbet and RORγt in CD4^+^FoxP3^-^live cells in adhered (**c**) and migrated (**d**) populations. Data were presented with mean ± SD, with triplicates in each condition. *P < 0.05, **P < 0.01, ***P < 0.001, using a two-tailed unpaired Student’s *t* test

To assess whether human lymphocyte migration was affected by GW559090, activated human PBMCs were pre-treated with GW559090 and assayed for their ability to migrate across inflamed HMEC monolayers grown on a fibronectin-coated matrix. Cells retained their viability for 18 h treated with 1, 10, 100, and 1000 μg/ml GW559090 (data not shown). Cell numbers post treatment with GW559090 in non-adherent (N), adhered (A), and migrated (M) populations relative to saline (Sal) were calculated (Fig. 5a), and demonstrated that numbers adhering or migrated did not change significantly with treatment. However, the percentages of cells that migrated were significantly decreased if pre-treated with at least 100 μg/ml GW559090 (Fig. 5b). The CD4^+^ T cell subsets in the non-adherent, adhered, and migrated populations were further evaluated by flow cytometry and a suppression of Th17 cells adhering to activated endothelium was obtained by pretreating with 1000 μg/ml GW559090 (Fig. 5c, d).

## Discussion

In this study, we report a significant attenuation of disease by the small-molecule inhibitor GW559090 when given topically. Disease was significantly reduced clinically, which  correlated with a reduction in retinal Th17 cells and a proportional Th1 cell increase. One explanation could be that the treatment accelerated the passage of the Th17 cells to an ex-Th17 cell phenotype. However, the overall level of Th1 cells in this model is relatively low and these cells are thought to be playing a minor pathogenic role as evident by the low level of disease observed following GW559090 treatment. We also investigated the functional role of GW559090 and the α4β1-dependent mechanisms involved in leukocyte infiltration into the retinal tissues before and during EAU using GW559090 eyedrops. Furthermore, the functional role of α4β1 in human CD4^+^ T cell transmigration was determined in vitro.

Our novel findings were firstly that EAU can be therapeutically downregulated by a topical α4β1 inhibitor, as seen both in the levels of retinal inflammatory cell infiltration and in clinical severity scores. Both rat and human neutrophils, eosinophils, monocytes, and lymphocytes are known to express α4β1 [17]. This small-molecule inhibitor of α4β1 preferentially downregulated Th17 cells and inflammatory monocyte/macrophages, without affecting Treg or neutrophils in the retina. We also determined that α4β1 facilitated adhesion, but not migration, of human Th17 cells to endothelial cells in vitro. The in vitro study design only investigated the effect of GW559090 on the T cells and not on the endothelial cells. Our data suggested that the inhibitor could decrease T cell adhesion in vitro and potentially influence the disease outcome. The role of the endothelial cells will be a focus for future study.

There is evidence for adhesion molecule expression in uveitis affecting man, where histological investigations demonstrated an increase in adhesion molecule expression in uveitis tissue specimens [9, 34, 35]. Furthermore, there is indirect evidence for adhesion molecule upregulation in bloods from Behçet’s disease [36]. The results suggested that the increased integrin expression levels in blood can only be detected when there is an associated systemic inflammation, although there is currently a lack of knowledge regarding integrin levels expressed in eyes from uveitis patients with localized disease. In animal studies, VCAM-1, which is reported not to be expressed in healthy eyes, was increased but only after EAU had developed, both at the site of post-capillary venules and on the retinal microvascular endothelium [5, 12, 37]. Thus, blocking the VLA-4/VCAM-1 pathway appears to be a more specific way of preventing the activated leukocytes from crossing the BRB. This has also been validated by systemically administered VLA-4 inhibitors which attenuated EAU disease severity [16, 19]. Our study attempted to achieve a localized therapeutic effect, thereby avoiding the serious side effects for example, re-activation of JC virus occurred within CNS glial cells which have occurred in multiple sclerosis patients receiving intravenous natalizumab (a humanized anti-α4 integrin antibody) [11]. In patients with refractory uveitic macular edema, it has been reported that an ICAM-1 inhibitor, Efalizumab (Raptiva, Genentech, San Francisco, CA, USA), could achieve therapeutic effect. However, a frequent report of PML and neurological symptoms in patients who had received efalizumab chronically led to its use being discontinued [38].

Interestingly, in EAU mice, the results suggested that if treatment failed with 3 mg/ml or 30 mg/ml GW559090 eyedrops, the disease was just as severe as in the control group. It is not clear yet if this reflects an inhibition of T cell migration across the BRB. However, a pharmacokinetic study was not conducted, and therefore this finding could simply be explained by a dose response effect, where 3 mg/ml was at the lower end of efficacy and that the 30 mg/ml dose was in excess and resulted in a negative feedback loop. We did not see any toxicity with any of the doses either on the ocular surface or within the retina.

The effectiveness of GW559090 as a topical therapy to reach the posterior segment of the eye is currently unclear. The effect may be explained either by a direct penetration of GW559090 into the retina, or an indirect absorption into the peripheral circulation through Schlemm’s canal, or a direct inhibitory effect on peripheral T cells which prevented immunopathogenic T cells adhering to and crossing the BRB. During the development of uveitis, it is essential for lymphocytes entering the eye to first traverse the vascular endothelial cell wall and cross the BRB. This process involves adhesive interactions between the endothelial cells and lymphocytes, and their subsequent migration across the endothelium. It has been described that the migratory process is controlled by adhesion molecules which may be upregulated following cell activation and has been well characterized in CNS studies [30]. This was supported by evidence that VCAM-1 expression was only detected if the endothelium was stimulated with IFNγ in vitro [10, 14, 39]. In contrast, in the LFA-1/ICAM-1 axis, ICAM-1 is expressed at the luminal surface continuously at intercellular junctions between adjacent endothelial cells even when the endothelium was not inflamed [5, 40]. In a study investigating rat retinal endothelial cells co-cultured with mitogen-activated antigen-specific T cells, it was demonstrated that adhesion on CNS vascular EC was largely dependent on LFA-1/ICAM-1 whether expressed on active or inactive EC, and only a small portion was dependent on VLA-4/VCAM-1 when the EC was activated by IL-1β [13]. In an in vitro study using primary human brain endothelial cells co-cultured with polarized Th1 and Th2 cells originated from healthy donor cells stimulated with or without myelin basic protein, it was reported that the process was more dependent on LFA-1/ICAM-1 interactions, while blocking the VLA-4/VCAM-1 axis had no effect on trans-endothelial migration [40]. These reports indicated that LFA-1/ICAM-1 interactions are important in general cell migration; VLA-4/VCAM-1 interactions are only involved in specific inflammatory migratory conditions. Our results agreed with the finding that blocking VLA-4 did not affect migrating cells, but the adhesion of CD4^+^ T cell subsets. Our results are limited by using healthy human PBMCs activated nonspecifically with PHA. The fact that GW559090 functionally reduced adhesion but not migration of Th17 cells could perhaps be due to their not being retinal antigen-specific and therefore were less migratory.

Under various inflammatory conditions, LFA-1/ICAM-1 and VLA-4/VCAM-1 paired axes may participate in lymphocyte migration differently. Our results suggested that individual CD4^+^ T cell subsets possess differential abilities with respect to their trans-endothelial migration, despite expressing a similar increased VLA-4 expression profile in all CD4^+^ T cell subsets within the retina and dLNs. This is supported by a previous CNS study in which the percentage of healthy peripheral blood-derived Th1 and Th2 cells expressed similar levels of VLA-4 and LFA-1 before and after migration across adult brain-derived endothelium in vitro. However, Th2 cells were more potent for migration than Th1 cells, even in the presence of an inhibitory ICAM-1 antibody [40]. In another study investigating VLA-4 on CD4^+^ T cells in experimental autoimmune encephalitis (EAE), it was reported that Th1 cells preferentially infiltrate the spinal cord via a VLA-4-mediated mechanism, whereas the entry of Th17 cells into the brain parenchyma occurred in the absence of VLA-4 integrins but was dependent on the expression of LFA-1 [41]. This conflicts with our findings but might reflect local barrier-specific differences. These results could also reflect tissue-specific differences in chemokine and chemokine receptor interactions in different autoinflammatory conditions. Crane et al. further supported this concept by reporting that CCR5 plays an important migratory role for antigen-specific Th1-polarized cells entering the BRB and into the retina during an inflammatory reaction rather than for rolling on the retinal vasculature during the initial stages of cell recruitment [42].

We discovered that by treating with GW559090 at the same time as inducing EAU, specific CD4^+^ T cells were blocked from crossing the BRB, thus preventing structural damage. In contrast, by administering GW559090 only after signs of EAU were already detectable, it appeared to prevent further cell infiltration, and low levels of disease could still be observed. It has been proposed in a time course study of EAU in B10.RIII mice that leukocytes cross the BRB at the level of the small venules, utilize selectin, and ICAM-1 before the disease appears and then utilize VCAM-1 and PECAM-1 when the disease starts to manifest, and that these conditions were observed for EAU but not in ovalbumin-immunized mice [5]. In our study, by blocking VLA-4 preventively, the disease severity was reduced, indicating low levels of adhesion molecule upregulation were associated with a decreased severity of disease. When blocking VLA-4 only after EAU had developed, a few lymphocytes might utilize other adhesion molecules infiltrating the BRB, which could explain why the disease was not fully ablated. The interaction between VLA-4/VCAM-1 is not a sole event. It was reported for human T cells that VLA-4 integrin/VCAM-1 interaction could activate LFA-1 integrin-mediated adhesion to ICAM-1 [43]. Inhibition of both pathways systemically exerted a synergistic effect in EAU [19]. However, we did not have the opportunity to test these inhibitors in our study.

As it has been shown that, apart from CD4^+^ T cells, other cells express VLA-4. We investigated retinal myeloid cells and found that if the α4β1inhibitor GW559090 was given therapeutically, a decrease of Iba1-expressing myeloid cells within the retinae and vitreous cavity and a reduction in Ly6C^+^ inflammatory monocytes/macrophages could be observed. Given that macrophages and dendritic cells were not detectable in CFA controls (data not shown), it is likely that the decrease in Ly6C^+^ inflammatory monocyte/macrophage was due to an inhibition of cell infiltration into the retina by GW559090 treatment. It has been reported that Ly6C^+^ inflammatory monocyte/macrophages appeared in EAU eyes a week post immunization and are involved in the chronic tissue damage seen in the eye during the course of uveitis [44, 45]. Activated macrophages have been demonstrated to use VLA-4 adhesion molecules as their primary integrin for in vitro CNS immigration [46]. It has been reported that early introduction of anti-VLA-4 mAb systemically can prevent CD45^hi^CD11b^+^ activated microglia/macrophages infiltrating the CNS along with targeting T cell infiltration to achieve a reduction in clinical severity in EAE [47]. Our study is the first to report that following GW559090, the Ly6C^+^ inflammatory monocyte/macrophages were abrogated in the retina, further supporting the CNS studies and suggesting its role to prevent further tissue damage.

## Conclusion

In conclusion, topical GW559090 significantly ameliorated CD4^+^ T cell infiltration into the eye, especially Th17 cells, without affecting systemic CD4^+^ T cell levels. The results were further supported by in vitro studies, showing that Th17 cells were specifically suppressed with respect to their adhesion to endothelial cells by GW559090. In addition, Ly6C^+^ inflammatory monocyte/macrophages in the eye were also ameliorated. α4β1 integrin-directed inhibition could therefore be of potential use in Th17-dominant uveitis disease.

## Supplementary Information

**Additional file 1.** Supplementary Fig.1 a Representative gating strategy to identify CD4^+^ T cell subsets in the retina for Fig.3. CD4^+^ T cells were first gated for single cells and live cells, and then for their expression of CD4. Within the CD4^+^ cell region, Th1 and Th17 were distinguished by intracellular expression of IFNγ and IL-17A respectively, within the FoxP3^-^ region. b Representative gating strategy to identify myeloid cell subsets within the retina for Fig. 4. Single live cells were gated for CD45 firstly, CD11b and CD11c, then CD64, Ly6G, and finally Ly6C.

**Additional file 2.** Supplementary Fig. 2 Representative flow cytometry figure depicting VLA-4 expression in different CD4^+^ T cell subsets in healthy control (Ctrl) and EAU eye.

## Data Availability

All relevant data generated or analyzed during this study are included in this published article.

## Abbreviations

A: Adhered
ARVO: Association for Research in Vision and Ophthalmology
BRB: Blood-retinal barrier
CNS: Central nervous system
EAU: Experimental autoimmune uveitis
EAE: Experimental autoimmune encephalitis
dLNs: Draining lymph nodes
ICAM-1: Intercellular Adhesion Molecule-1
IRBP: Interphotoreceptor retinoid-binding protein
LFA-1: Leucocyte functional antigen-l
M: Migrated
N: Non-adherent
PBS: Phosphate-buffered saline
PML: Progressive multifocal leukoencephalopathy
Sal: Saline
VCAM-1: Vascular cell adhesion molecule-1
VLA-4: Very late activation antigen-4
