# Supplementary figures and images for: Small-molecule antagonist of VLA-4 (GW559090) attenuated neuro-inflammation by targeting Th17 cell trafficking across the blood-retinal barrier in experimental autoimmune uveitis
Source: J Neuroinflammation. 2021 Feb 18;18:49. doi: 10.1186/s12974-021-02080-8 (PMC7893745; doi:10.1186/s12974-021-02080-8)

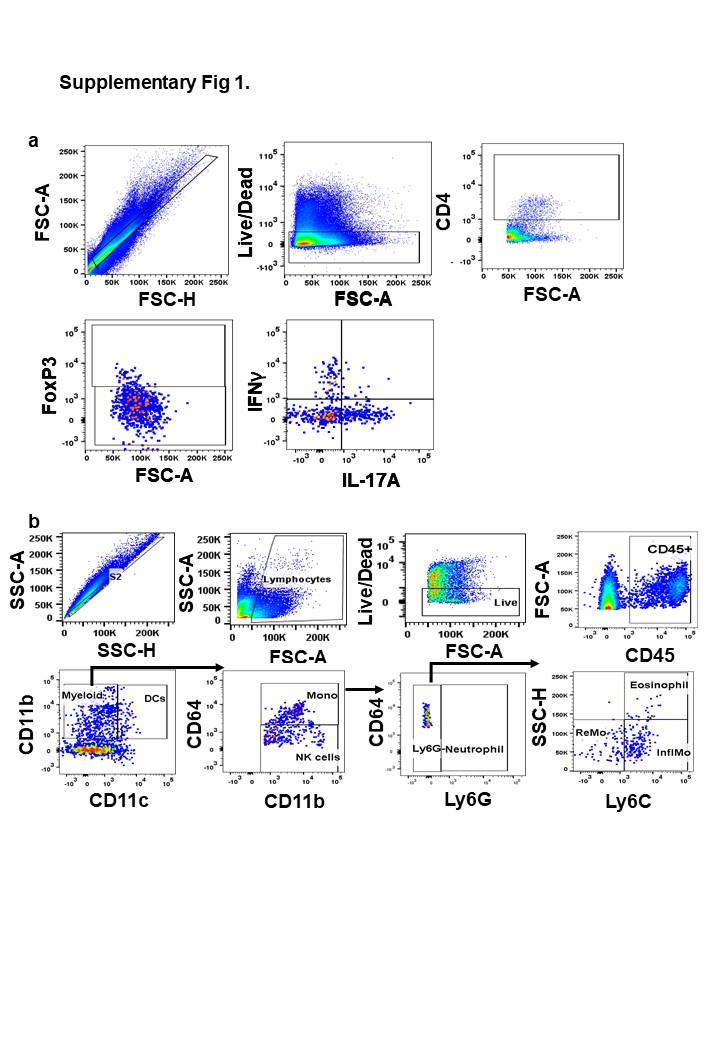

Supplement: Supplementary file 1 — Additional file 1. Supplementary Fig.1 a Representative gating strategy to identify CD4+ T cell subsets in the retina for Fig.3. CD4+ T cells were first gated for single cells and live cells, and then for their expression of CD4. Within the CD4+ cell region, Th1 and Th17 were distinguished by intracellular expression of IFNγ and IL-17A respectively, within the FoxP3- region. b Representative gating strategy to identify myeloid cell subsets within the retina for Fig. 4. Single live cells were gated for CD45 firstly, CD11b and CD11c, then CD64, Ly6G, and finally Ly6C. [file 12974_2021_2080_MOESM1_ESM.jpg]

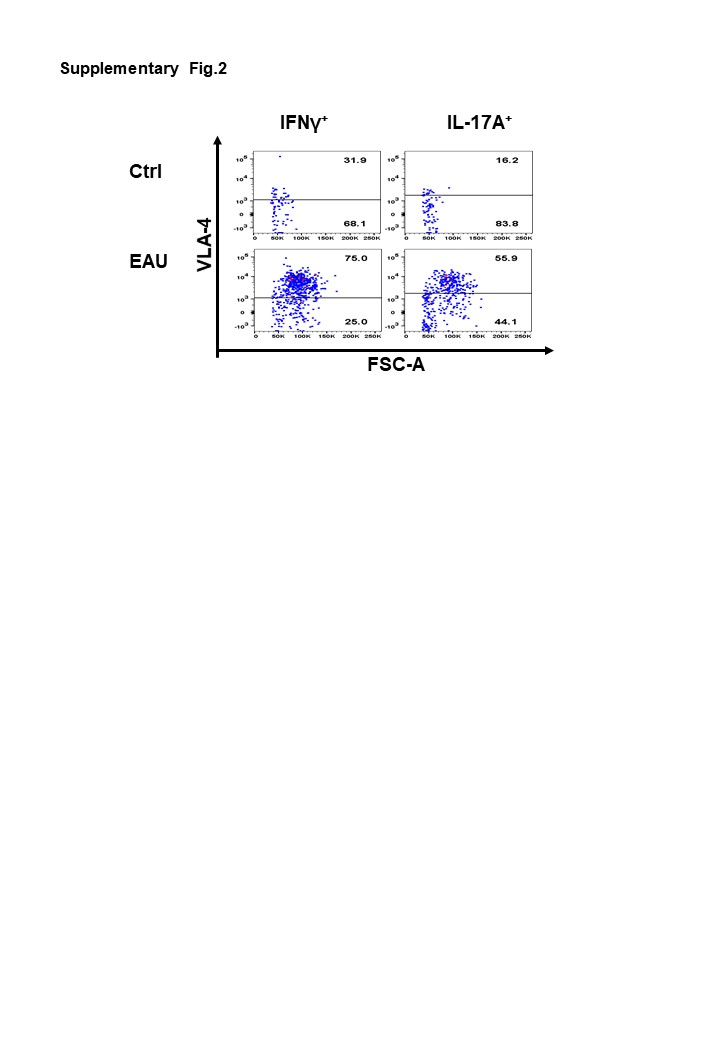

Supplement: Supplementary file 2 — Additional file 2. Supplementary Fig. 2 Representative flow cytometry figure depicting VLA-4 expression in different CD4+ T cell subsets in healthy control (Ctrl) and EAU eye. [file 12974_2021_2080_MOESM2_ESM.jpg]
